# Structural Equation Modeling of In silico Perturbations

**DOI:** 10.3389/fgene.2021.727532

**Published:** 2021-11-24

**Authors:** Jianying Li, Pierre R. Bushel, Lin Lin, Kevin Day, Tianyuan Wang, Francesco J. DeMayo, San-Pin Wu, Jian-Liang Li

**Affiliations:** ^1^ Integrative Bioinformatics, Epigenetics and Stem Cell Biology Laboratory, Division of Intramural Research, National Institute of Environmental Health Sciences, Durham, NC, United States; ^2^ Kelly Government Solutions, Durham, NC, United States; ^3^ Massive Genome Informatics Group, National Institute of Environmental Health Sciences, Durham, NC, United States; ^4^ Biostatistics and Computational Biology Branch, Division of Intramural Research, National Institute of Environmental Health Sciences, Durham, NC, United States; ^5^ Department of Family Health Care Nursing, University of California, San Francisco, San Francisco, CA, United States; ^6^ Reproductive and Developmental Biology Laboratory, National Institute of Environmental Health Sciences, Durham, NC, United States; ^7^ Duke University, Durham, NC, United States

**Keywords:** structural equation modeling, gene expression, *In silico* perturbation, molecular interaction, R Shiny

## Abstract

Gene expression is controlled by multiple regulators and their interactions. Data from genome-wide gene expression assays can be used to estimate molecular activities of regulators within a model organism and extrapolate them to biological processes in humans. This approach is valuable in studies to better understand complex human biological systems which may be involved in diseases and hence, have potential clinical relevance. In order to achieve this, it is necessary to infer gene interactions that are not directly observed (i.e. latent or hidden) by way of structural equation modeling (SEM) on the expression levels or activities of the downstream targets of regulator genes. Here we developed an R Shiny application, termed “Structural Equation Modeling of In silico Perturbations (SEMIPs)” to compute a two-sided t-statistic (T-score) from analysis of gene expression data, as a surrogate to gene activity in a given human specimen. SEMIPs can be used in either correlational studies between outcome variables of interest or subsequent model fitting on multiple variables. This application implements a 3-node SEM model that consists of two upstream regulators as input variables and one downstream reporter as an outcome variable to examine the significance of interactions among these variables. SEMIPs enables scientists to investigate gene interactions among three variables through computational and mathematical modeling (i.e. *in silico*). In a case study using SEMIPs, we have shown that putative direct downstream genes of the GATA Binding Protein 2 (GATA2) transcription factor are sufficient to infer its activities *in silico* for the conserved progesterone receptor (PGR)-GATA2-SRY-box transcription factor 17 (SOX17) genetic network in the human uterine endometrium.

## Introduction

While gene expression data in public repositories provides a valuable resource for investigators to infer regulatory processes (Edgar et al., 2002), the causal or unobserved (i.e. latent) gene interactions are a challenge to detect. Moreover, the extrapolation of biological processes and regulatory networks from experimental model systems to humans in order to infer causation of diseases can be a formidable task. Fortunately, genome-wide gene expression assays on human specimens capture observations of correlations among the gene expression levels as well as between RNA abundances and phenotypic outputs. These gene expression assays can also determine the downstream targets of a factor of interest in model systems that are relevant to the particular type of human specimen via genetic or pharmacological perturbations (Koot et al., 2016). The resulting gene signature, comprised from the expression of these downstream target genes in response to a perturbation, could unbiasedly serve as a surrogate of the activity of the factor of interest in a given context. Assuming that gene activities and biological functions are preserved between humans and relevant model systems, the degree of similarity between the gene expression signature of the regulator of interest and the model organism’s gene expression profile can be quantitatively estimated by a T-score calculation from t-tests of gene expression data to represent gene regulatory activities in the targeted organism (Creighton et al., 2008; Creighton et al., 2009; Luo et al., 2009; Qin et al., 2014). This scoring system has been employed to establish correlations between the prognosis outcome and manifestation of activities of the factor of interest in corresponding tumors (Creighton et al., 2008; Creighton et al., 2009; Luo et al., 2009; Qin et al., 2013; Qin et al., 2014). The T-score calculation has also been utilized to determine the association among activities of factors of interest or between the activities of an upstream regulator and levels of its downstream targets within a set of human specimens (Wu et al., 2015; Rubel et al., 2016). Results of these studies demonstrated applications of such a surrogate score of molecular activities in investigation of gene functions and inference of regulatory processes (Grace 2006).

To determine the relationships among multiple variables, structural equation modeling (SEM) is a statistical technique to indicate the strength of influence among variables (Edgar et al., 2002; Grace 2006). We were motivated to develop a Structural Equation Modeling of In silico Perturbations (SEMIPs) R Shiny application (app) to facilitate casual inference of gene regulatory processes, especially on multifactorial impacts on outcome variables concurrently. SEMIPs enables quantification of a projected activity metric (T-score) and allows users to fit desired SEM models using gene variables of interest. For hypothesis generation purpose, SEMIPs provides two different bootstrap random sampling procedures (elimination with or without replacement) to test the significance of the model (Creighton et al., 2008). Previously, the T-score and SEM were applied to gene expression data to evaluate gene interactions that regulate the progesterone signaling pathway in the mouse uterus and infer gene regulation processes in human uterine specimens (Rubel et al., 2016). SEMIPs streamlines this process and allows scientists to perform the computations and analyses through a user-friendly interface.

## Materials and Methods

### Overview of SEMIPs

**FIGURE 1:**
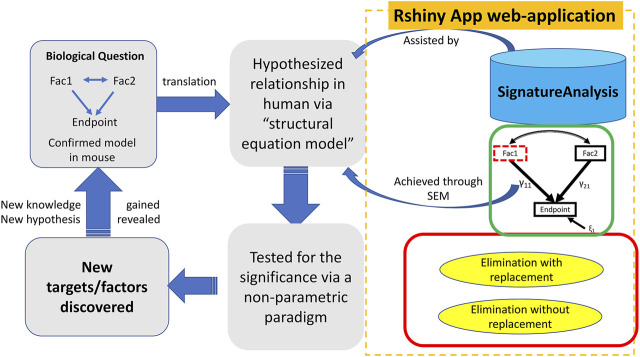
The workflow and application of SEMIPs. The left four rectangles and arrows indicate our hypothesis testing and generation schema; the components bounded by the dotted orange rectangle are features provided in the Rshiny App web-application. A biological hypothesis is tested in a model system (i.e. mouse) on relationship between two interacting factors (Fac1 & Fac2) and their endpoint through a 3-node SEM model indicated by the green rectangle. The hypothesis is translated to another species (i.e., human in our research) *via* T-score computation (represented by the upper blue arrow noted as “assisted by”) and verified with the SEM model (represented by the lower blue arrow noted as “achieved through SEM”). This process is accomplished with our R Shiny app indicated by two curved arrows. *γ*_11_ and *γ*_21_ are correlation coefficients and ξ_1_ is the model residual. The two-class bootstrap resampling is shown in the red rectangle box. Hypothesis generating and exploring steps are explained by the bottom two rectangles.

The SEMIPs R Shiny app allows users to compute T-scores from gene expression data to infer the activities of genes of interest in a quantitative manner. Shown in Figure 1, the SEMIPs app (highlighted in the orange dotted rectangle) facilitates the hypothesis generation and testing framework. This app also provides a 3-node model fitting function using the SEM to test the joint regulation of a target gene by two upstream regulators *in silico*. In addition, for hypothesis generation purposes, a two-class bootstrap method, elimination with replacement or elimination without replacement, is included in the app to examine the impact of removing genes that belong to the same signaling cascade from the downstream targets of the gene of interest.

### T-Score Calculation

The T-score calculation requires the input of two components: 1) a normalized gene expression matrix of the human specimens and 2) a gene signature of the factor of interest from the model organism. To generate the normalized gene expression matrix of human tissues (microarray or RNAseq data) the expression values of each gene are centered to the median across all samples. If a gene has multiple probes or transcripts, the probe/transcript with the highest variation (i.e. the standard deviation) was chosen to represent that gene. The gene signature is first determined by identifying downstream target genes whose RNA abundance are associated with the levels of the upstream regulator. The downstream targets are further subgrouped based on the positive (up-regulated signature) or negative (down-regulated signature) correlations on the RNA abundance between the upstream regulator and the downstream targets. The T-score is then calculated based on the following formula:

T-score=d∗TINV(p,df)

where *d* = 1 if the average expressions of homologous genes of up-regulated signature genes is larger than the average expressions of homologous genes of down-regulated signature genes, otherwise *d* = -1; TINV is the function for the two-tailed inverse of the *t*-distribution; *p* is the *p*-value from two-tailed *t*-test of the expressions of homologous genes of up-regulated signature genes and the expressions of homologous genes of down-regulated signature genes assuming equal variance; and *df* is the degrees of freedom (the total number of the homologous genes of signature genes minus 2).

**FIGURE 2:**
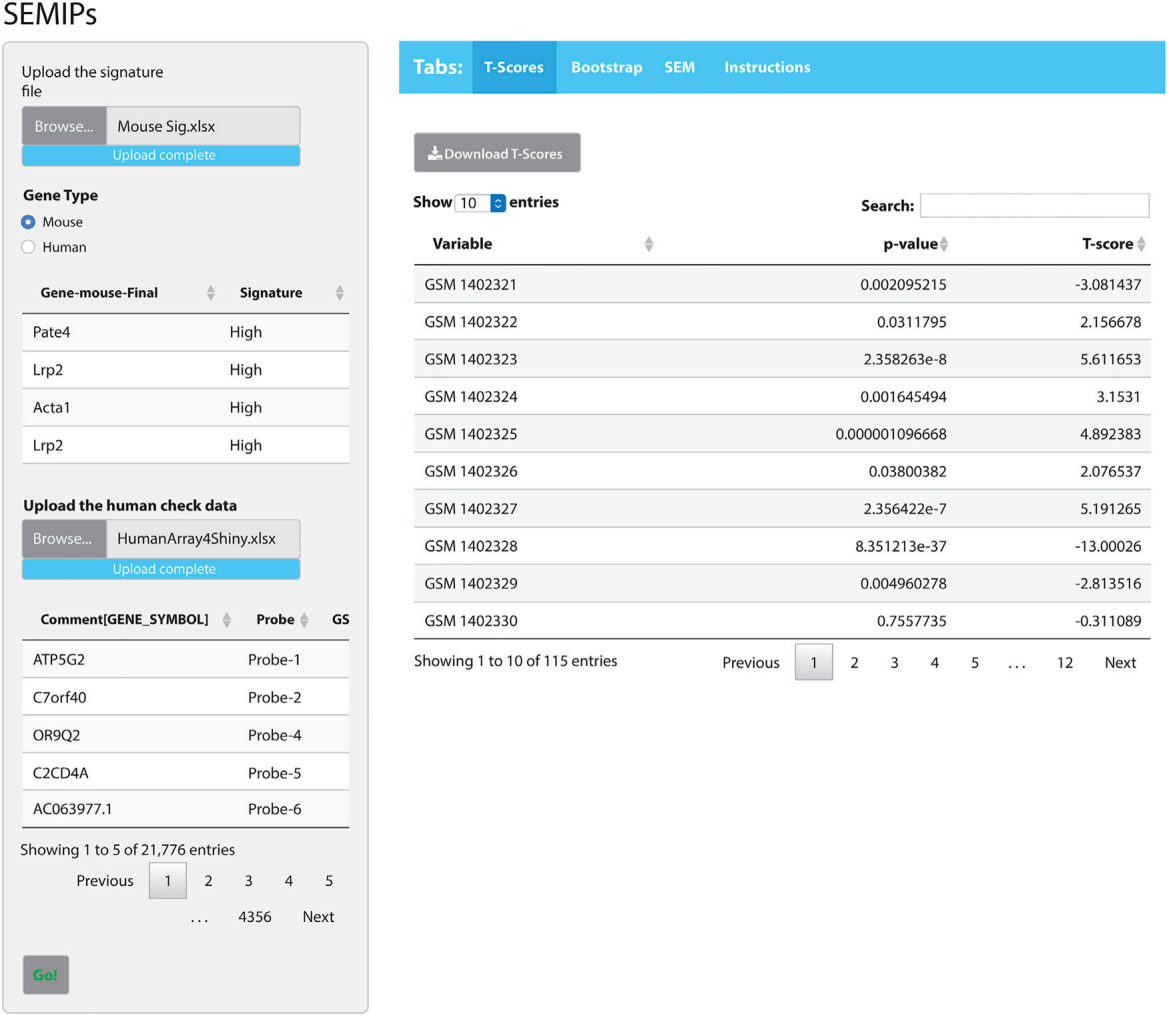
The SEMIPs user interface. The main panel contains four tabs: “T-Scores”, “Bootstrap”, “SEM”, and “Instructions”. The right panel shows the screen when the “T-Scores” tab is selected and generated. In the left panel, the application accepts two inputs: 1) a list of signatures (in Entrez gene symbol format) and 2) a data matrix of expression measurement with the top lines shown for viewing. The green “Go!” button is clicked to launch the T-score generation and grayed out to denote the process is running. The first 10 rows of the T-scores matrix are shown; however, the entire matrix can be downloaded by clicking the “Download T-Scores” button.

The hypothesis generation relies on results obtained from a perturbation of an animal model system, then projects into human or other animal model systems when either the direct perturbation is not possible or the variables of interest are not directly measurable (Rubel et al., 2016). The SEMIPs R Shiny app provides a user-friendly way to calculate the T-scores via the tab labeled “T-Scores” as shown in Figure 2. The application will conduct the analysis and produce inferred activity results that can be used in subsequent downstream analyses. Users can use the “T-scores” calculation feature to calculate T-scores from any custom prepared gene lists obtained from microarray or RNAseq experiment either in mouse gene symbols or human gene symbols (shown in Supplementary Figure S1).

### Structural Equation Modeling

The second feature of the SEMIPs app is the SEM. We implemented the SEM using the lavaan R package (Rosseel, 2012) to provide a 3-node model fitting function to test the joint regulation of a target gene by two upstream regulators *in silico*. T-scores and/or normalized RNA levels of two upstream regulators are the two input variables, while the outcome variable is the value of the RNA expression level of a chosen downstream reporter gene that is expected to be regulated by the two upstream regulators. The SEM fit can be assessed using various criteria, including the root mean square error of approximation (RMSEA), along with a 90% confidence interval, the Comparative Fit Index (CFI), the Tucker-Lewis Fit Index (TLI), and the standard root mean square residual (SRMR). For RMSEA, the general rule of thumb is that values < 0.05 indicate close fit, values between 0.05 and 0.10 indicate marginal fit, and values > 0.10 indicate poor fit (MacCallum et al., 1996). For both the CFI and the TLI, a value of 1 indicates perfect fit, and the general rule of thumb is that values > 0.90 indicate adequate fit (Hu and Bentler 1998; Hu and Bentler 1999). Also, SRMR values < 0.08 indicate a very good fit between the model and the data.

The app comes packaged with a sample data file “app_installation_dir/dataSEM/sampleDAT.txt”. When the SEM tab is selected (Figure 2), this data file will be loaded and users can select three variables from the drop-down list to test the SEM model. The SEMIPs app also provides a data file template “app_installation_dir/dataSEM/_sampleDAT.txt” that users can modify and save as “sampleDAT.txt” to overwrite the default data. As a result, the users’ data will be loaded when the app is launched subsequently. Users can save the modeling figures and all fitting statistics from the app.

### Bootstrap Simulation

**FIGURE 3:**
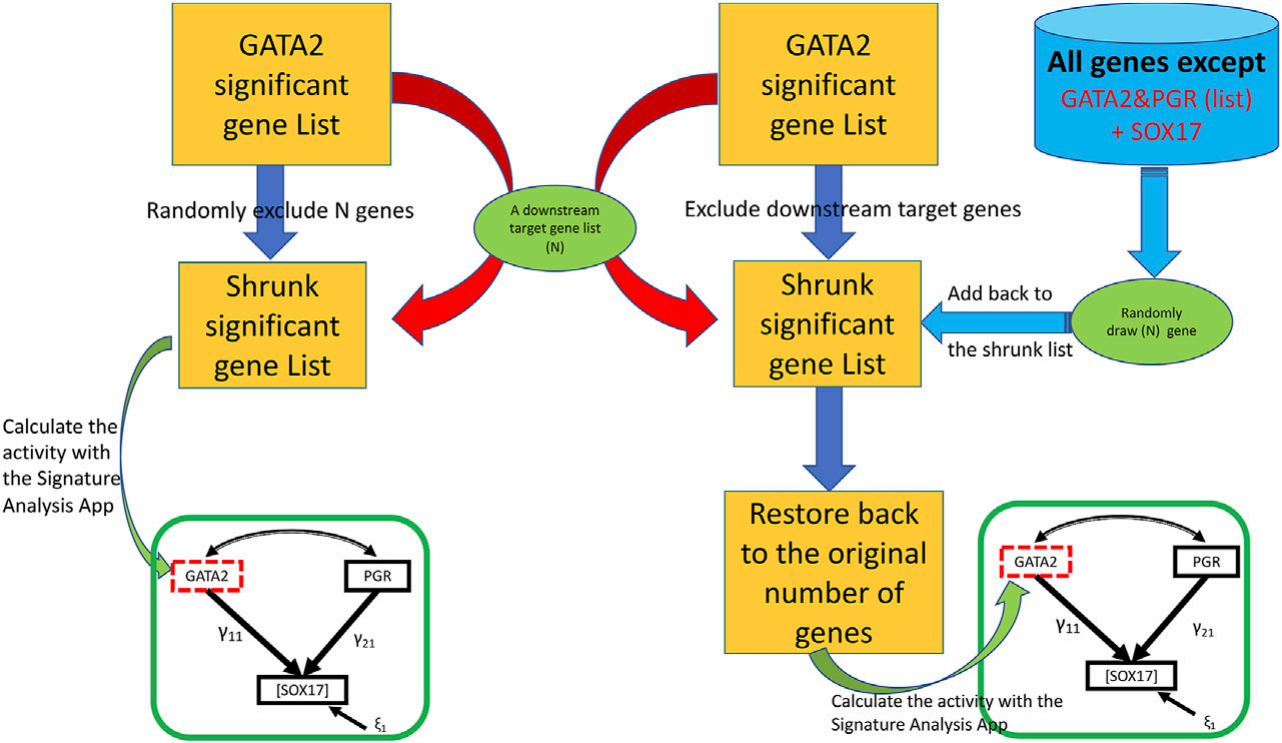
A two-class bootstrap resampling (elimination with or without replacement) simulation. From the initial GATA2 significant gene list represented as the yellow rectangle, the downstream target genes (“N”) are eliminated in the without replacement simulation (left side) giving rise to the shrunk significant gene list represented by a smaller yellow rectangle; in the elimination with replacement simulation (right side), the same number of genes as that of the targeted subset of genes (“N”) are eliminated giving rise to the shrunk significant gene list, and then restored back to the original size by adding back randomly draw (“N”) represented by the far right green oval from the gene pool represented by the blue cylinder. In the elimination without replacement, the resulting shrunken GATA2 gene list is used to calculate the T-scores, then fed into the SEM model indicated by the green rectangle. In the elimination with replacement, the restored gene list is used to calculate the T-scores, then fed into the SEM model. The simulation can be repeated for a large “number of bootstraps” to generate a non-parametric distribution for statistics significance.

The third feature (the “Bootstrap” tab shown in Figure 2) assesses the potential impact from a perturbation on the proposed genetic network such as removing a downstream molecular pathway or the gene signature of a downstream effector from the upstream regulator. We implemented a two-class (elimination with or without replacement) bootstrap resampling for statistical inference (Figure 3), which eliminates unrelated signatures and provides statistical significance to the SEM fitting. For this feature, it is assumed that the user has successfully run a T-score analysis. The user also need to enter the signatures associated with the downstream system of interest to evaluate. To improve the rigor of the statistical test, it is recommended to run the bootstrap a minimum of 1,000 iterations to potentially obtain a *p*-value as small as 0.001. Since this feature involves bootstrapping simulation, it requires multicore hardware and can take longer to complete the computations depending on how many iterations the user choose.

### Sample Data

The SEMIPs app is packaged with four test datasets and data templates for the user to test the app and further modify to suit their own study. The test data are located at “app_installation_dir/testData”.

### Hardware and Software Requirement

SEMIPs was written in R with the Shiny package (Xie, 2014) that is known for its light weight web development framework with shiny-related features. The lavaan package (Rosseel, 2012) was used for the SEM. Dependent packages will be instansiated or they need to be installed if not already available. The application requires modern multicore CPUs for the backend parallel processes. SEMIPs was developed under Linux CentOS7 and has been successfully tested on MacOS (v. 10.14.6) and Windows10. To install and run this application, users can follow the detailed instructions provided in the README. txt file. The SEMIPs Shiny app and source code are freely available at https://github.com/NIEHS/SEMIPs under the MIT license.

## Results

### An Integrated Hypothesis Generation and Testing Framework

As shown in Figure 1, the SEMIPs workflow depicts a genetic interaction among genes of interest that is initially revealed in a model system and then tested for its manifestation in human specimens via model fitting. SEMIPs is designed to test concurrent contributions of regulatory effects of two upstream regulators “Fac1” and “Fac2” to the expression of a downstream reporter gene “Endpoint”. Meanwhile, two-directional interactions between the two upstream regulators are examined. Under this structure, users can test the relationships among the gene expression levels of all three variables. If a hypothesis involves testing of molecular activities of two upstream regulators, gene signatures of the upstream regulators are first projected to a gene expression matrix of human specimens of interest (e.g., an expression dataset that are derived from human biopsies) through the T-score calculation function. The resulting T-scores will serve as the surrogate molecular activities to test for the manifestation of the proposed genetic network in human specimens via model fitting.

For hypothesis generation purposes, a subset of genes that are associated with pathways of interest or downstream effectors can be removed from the upstream regulator’s gene signature as an *in silico* perturbation to infer the potential impact of losing the downstream signaling on the activities of the upstream regulator (Creighton et al., 2008). Based on the SEM model, a presumed relationship can be tested in humans by determining the significance of the inference via a non-parametric bootstrap resampling framework. Any resulting perturbed pathways that are significant would help to prioritize experiments in model systems. These workflow steps are shown within the dotted rectangle on the right side of Figure 1 with the three major features implemented in the SEMIPs app as the function tabs (Figure 2).

### T-Score Calculation to Aid in Translational Research

The T-score was employed to project molecular activities of a gene of interest from a model system experiment to human specimens where a perturbation was not directly applicable (Creighton et al., 2008; Creighton et al., 2009; Luo et al., 2009; Qin et al., 2014). In a model system, the subjects are randomly assigned into two groups, where one group will receive “placebo” and/or no treatment and the other group will receive a treatment as a perturbation. Experimental measurements will be properly collected from both groups (i.e., gene expression profiles from a genome-wide gene expression experiment). Significantly changed genes/probes (signatures) will be obtained from this analysis according to pre-determined thresholds followed by a statistical analysis with directionality (up/down regulation). Such a list of genes/probes are deemed collectively as the “gene signature” of biological responses to a particular perturbation in a given context such as cell or tissue types of interest. In addition, these downstream target genes of the perturbed system are referred to as “signature genes”. This gene signature information will be projected into the human specimen of interest bearing the assumption that the biological functions of the genes of interest are conserved between the chosen model system and the human specimen.

In the gene expression dataset (i.e., human) of which the molecular activities of the factor of interest on individual samples are to be estimated, the orthologs of the signature genes are first identified and grouped based on the directionality of the signature genes. The T-scores of individual samples in the dataset are derived from a *t*-test between the two groups of measurements to compose a single number as a quantitative surrogate of molecular activities of interest. Samples with T-scores larger than 0, which share a similar signature gene expression profile from the model system, were classified as having gene activities and vice versa.

As an example of how to use the SEMIPs app, we provide: 1) a list of a human gene signature in Entrez gene symbol format (Human Sig. xlsx) and 2) a data matrix of human gene expression profiles (HumanArray2Shiny.xlsx) located under “/app_installation_dir/testData/t-score/“. Once the data files are uploaded, the top few lines of data are visible for preview (Figure 2). As an additional example, we also provide the mouse signature genes (Mouse Sig. xlsx) and homologus human signature file. After the species is properly matched by selection, the T-scores will be calculated by clicking the green “Go!” button. The top 10 rows of the T-scores will be shown for preview. The usercan download the T-scores for further analysis. Since the T-scores are calculated from a two-side *t*-test, the corresponding *p*-values are also reported (the second column in T-scores results shown in Figure 2).

### Structural Equation Modeling

The impact of genetic interactions among regulators on downstream target genes is often tested by simultaneous manipulations on levels or activities of the regulators in a model system. The SEMIPs app takes advantage of publicly available or existing gene expression information to examine such potential interactions *in silico* by SEM. SEMIPs supports the testing of hypotheses in which two upstream regulators (“Fac1” and “Fac2”) concurrently regulate the levels of one downstream reporter gene (Endpoint) in a 3-node model (Figure 1). The input variables for upstream regulators can be either the gene expression levels or the molecular activities in T-score format. Our current SEM model tests both upstream regulators in relation to the “endpoint”, where γ_11_ and γ_21_ are the coefficients in the regression model and ε_1_ is the residual (Figure 1). Once the SEM tab is selected, the default data will be loaded and all features are available for the user to choose from the drop-down windows (Figure 2). The two exogenous variables (Fac1 & Fac2) are hypothesized as “causal factors” in the SEM model and one endogenous variable (Endpoint) as the “effect” (Figure 1). The app reports model fitting statistics and the three-node SEM figure both of which can be downloaded. This feature also allows users to test a separate system by uploading their relevant dataset. The dataset requires the same format as the example data. Results derived from SEMIPs could possibly aid in the prioritization of wet lab experiments and the establishment of clinical relevance.

### Two-Class Bootstrap Simulation

Biological signaling is often transduced by a cascade of downstream effectors in a hierarchical manner. The gene signature of an upstream regulator is usually a collective presentation of activities of multiple downstream effectors whose mRNA abundance may or may not be altered upon stimulations. *In silico* dissection of the contribution of effectors to the upstream regulators’ effect has been utilized previously by removing genes that reflect the effector’s activities from the upstream regulator’s gene signature (Creighton et al., 2008). In the SEMIPs app, genes that are associated with biochemical pathways, or belong to the downstream effector’s gene signature, can be tested with two-class bootstrap resampling (elimination with or without replacement) for statistical significance (Figure 3). In the “elimination without replacement” process, we attempt to eliminate the same number (N) of irrelevant genes, then continue with the following SEM modeling steps etc. On the other hand, in the “elimination with replacement” process, we first eliminate “actual downstream target genes (N)”, and then randomly select the same number of “irrelevant genes” from the pool (indicated by the blue cylinder as shown in Figure 1) and put them back into the shrunken list to restore back to the same number of genes as the “GATA significant gene list” followed by the following SEM modeling steps.

The app package comes with four downstream gene sets to test the boostrap resampling. Under the “Bootstrap” tab, the user can load these gene sets and run the bootstrap simulation analysis. The impact on the downstream system can be assessed by either elimination without replacement or with replacement. To ensure the rigor of the statistical test, it is recommended to run the bootstrap a minimum of 1,000 times. Depending on the hardware configuration, this analysis can take a considerable amount of time. Users can download the zipped results after the analysis is completed (shown in Supplementary Figure S2). The results derived from this analysis could potentially serve as a rationale to further genetic or pharmacological experiments.

### A Use Case Application

**FIGURE 4:**
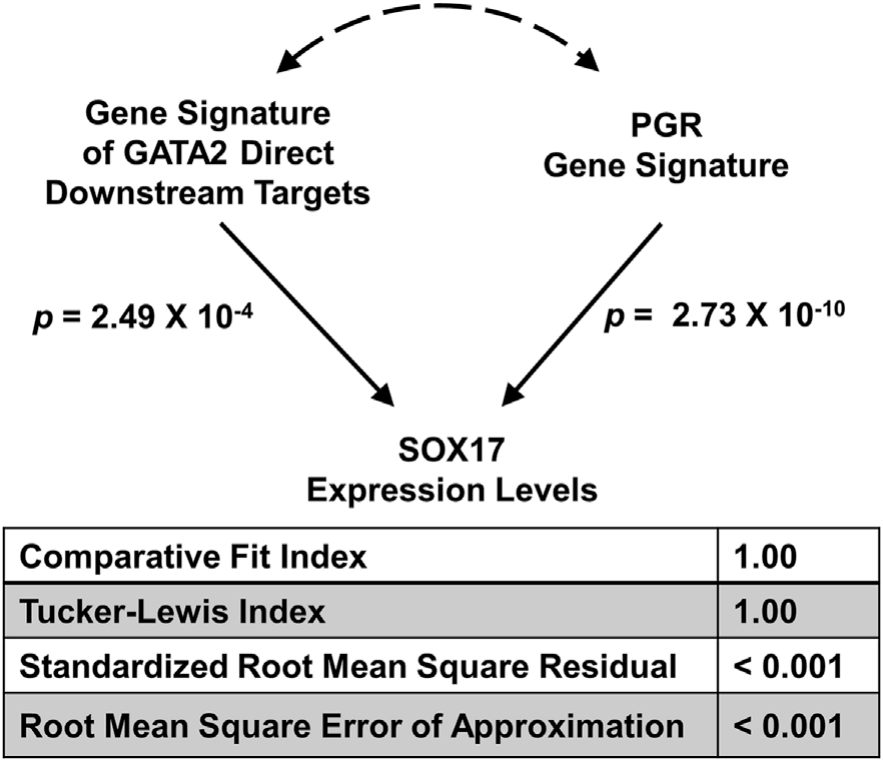
Major model fitting statistics for the joint regulation of the SOX17 gene expression levels by GATA2 and PGR activities in the GEO accession: GSE58144 dataset illustrated in the 3-node SEM. Two exogenous variables are “Gene Signature of GATA2 Direct Downstream Targets” and “PGR Gene Signature” respectively, and one endogenous variable is “SOX17 Expression Levels”.

Previously we demonstrated that the mouse gene signatures of GATA Binding Protein 2 (GATA2) and progesterone receptor (PGR) allow inference of the interaction between the two transcription factors for regulation of SRY-box transcription factor 17 (SOX17) expression in the human endometrial tissues (Rubel et al., 2016). The full GATA2 gene signature consists of both direct and indirect downstream genes of the transcription factor in the uterus (Rubel et al., 2016). Since GATA2 is a transcription factor that occupies *cis*-acting elements and confers genomic regulation activity, we hypothesize that the expression levels of GATA2’s direct downstream targets reflect its activities *in silico.* Here, a direct downstream target of GATA2 is defined as a GATA2-regulated gene with GATA2 genomic occupancy within the 2-kilobase vicinity of its transcription start site in the uterus (Gene Expression Omnibus (GEO) accession: GSE40659 (Rubel et al., 2016)). This stringent criterion led to the identification of a list of 634 genes (Supplementary Table S1), which is termed the “GATA2 direct signature”. The GATA2 activity, as represented by the GATA2 direct signature in a T-score, was quantified by the SEMIPs app from gene expression data of the endometrium tissue for each individual human subject (GEO accession: GSE58144 (Koot et al., 2016)). T-scores for the uterine GATA2 in all 115 patients were calculated by the app with the GATA2 direct signature and the data matrix of GEO accession: GSE58144 (Supplementary Table 2). Similarly, T-scores for the uterine PGR (termed the “PGR signature”) were obtained using the GEO accession: GSE39920 dataset (Rubel et al., 2016) on the same data matrix via the application’s T-score calculation function. To test whether the GATA2 direct signature fits the model of the 3-node PGR-GATA2-SOX17 genetic network, the application via the SEM tab, was fed with T-scores of the GATA2 direct signature and the PGR signature as exogenous variables, and the SOX17 expression levels as the endogenous variable. The analysis results show that given the GATA2 direct signature in place of the full gene signature, the model significantly fits the GEO accession: GSE58144 dataset with all proposed paths (Figure 4) and this model is considered not rejected by the human data. This finding suggests that the expression levels of the GATA2 direct downstream targets, a subset of the full GATA2 regulated genes, can serve *in silico* as surrogate reporters of the GATA2 activities in the human endometrium tissues. This supports the hypothesis that gene expression patterns of GATA2 direct downstream target genes are capable of reflecting GATA2’s activities in this context. Results of this analysis not only reduce the number of reporter genes for GATA2 activities to 634, but also implicate possibilities of a further reduction with additional filtering criteria on the gene list. A small and manageable panel of markers for GATA2 activities could serve as a future diagnostic tool for pregnancy failure (Diaz-Gimeno et al., 2011).

## Discussion

The SEMIPs R Shiny app offers an easy to use *in silico* perturbation testing system with several advantages. First, it has the capability of calculating gene activities using large datasets representative of biological systems. Second, it leverages the power of SEM to test the relationship among end points in a study and provides users with the flexibility for testing new hypotheses. Lastly, it integrates a non-parametric testing procedure for assessing statistical significance.

This app allows users quick assessments of genetic interactions and subsequent hypothesis generation without having to know computer programming or statistical modeling. Due to its simplicity in design, this app is limited to a 3-node model fitting. Models of higher complexity can be tested using the R package MplusAutomation that focuses on automating the SEM modeling which was originally implemented in the commercial software Mplus (Hallquist and Wiley 2018). MplusAutomation uses open-source R to mirror Mplus functionality and automates modeling three major aspects of latent variable modeling, including creating a group of models, running them in batches, and extracting the model fitting statistics. Our SEMIPs app is similar to MplusAutomation in that the SEM model is implemented but rather in R instead of Mplus for wide availability. We use the lavaan package, a highly credited/cited package in the research community since 2012 to implement the SEM model and extract all the statistics from the modeling output. The goal of SEMIPs is to provide a convenient and easy to use tool that bridges bioinformatic assessments and scientists who have minimum computation background for hypothesis generation and inferring biological processes across experimental systems. This is achieved by employing R shiny to render a user friendly web front end, as demonstrated in the manuscript.

Currently, the two-class bootstrap analysis can only be conducted separately. Integration of these into the SEMIPs methodology for formulation into a single test will be investigated for future design, development, and implementation. As noted in the manuscript and mentioned previously, the SEMIPs app has been adopted by wet lab researchers with a few papers published recently (Liu et al., 2019; Wetendorf et al., 2020). We hope that it can serve a wider research community to address additional scientific questions.

## Data Availability

Publicly available datasets were analyzed in this study. These datasets can be found here: https://www.ncbi.nlm.nih.gov/geo/query/acc.cgi?acc = GSE40659 https://www.ncbi.nlm.nih.gov/geo/query/acc.cgi?acc = GSE58144 https://www.ncbi.nlm.nih.gov/geo/query/acc.cgi?acc = GSE39920.
