# Supplementary material for: Structural Equation Modeling of In silico Perturbations
Source: Front Genet. 2021 Nov 24;12:727532. doi: 10.3389/fgene.2021.727532 (PMC8652139; doi:10.3389/fgene.2021.727532)
Supplement: Supplementary file 2 [file DataSheet1.pdf]

Structural Equation Modeling of In silico Perturbations

Jianying Li[1,2,3 †], Pierre R. Bushel[3,4 †], Lin Lin[5,6], Kevin Day[7], Tianyuan Wang[1,2], Francesco J. DeMayo[6], San-Pin Wu[6 *], and Jian-Liang Li[1 *]

[1] Integrative Bioinformatics, Epigenetics and Stem Cell Biology Laboratory, Division of Intramural Research, National Institute of Environmental Health Sciences, Research Triangle Park, NC 27709, USA

[2] Kelly Government Solutions, Research Triangle Park, NC 27709, USA

[3] Massive Genome Informatics Group, National Institute of Environmental Health Sciences, Research Triangle Park, NC 27709, USA

[4] Biostatistics and Computational Biology Branch, Division of Intramural Research, National Institute of Environmental Health Sciences, Research Triangle Park, NC 27709, USA

[5] Department of Family Health Care Nursing, University of California at San Francisco, San Francisco, CA 94143, USA

[6] Reproductive and Developmental Biology Laboratory, National Institute of Environmental Health Sciences, Research Triangle Park, NC 27709, USA

[7] Duke University, Durham NC 27708

[†]These authors have contributed equally to this work and share first authorship

*** Correspondence:**
Jian-Liang Li (jianliang.li@nih.gov) and San-Pin Wu (steve.wu@nih.gov)

Running Title: Structural Equation Modeling In silico

Keywords: Structural Equation Modeling, gene expression, *in silico* perturbation, molecular interaction, R, Shiny

Supplemental Methods

Two-class bootstrap simulation

The primary objective of selecting SEM in our research and fundamental advantage of SEM is to allow researchers to derive the relationship between variables of interest when these variables are not directly measurable. In the proposed SEMIPs method, we tested the relationship via a 3-node SEM model among three variables in a complex genomic system. Each of these variables can either be a regulator that regulates a group of downstream genes or a readout of impact from some upstream regulator. The a two-sided t-statistic, naminglyT-scores are calculated based on the direction of these upstream/downstream signatures(genes), and then used in the SEM modeling.

When we have a group of signatures (genes) obtained from an experiment (i.e. a KEGG pathway analysis in our paper, data comes with the SEMIPs application at "/app_installation_dir /testData/bootstrap/"), we are interested in finding out whether a regulator (upstream/downstream) is associated with a factor (i.e. GATA2 in our example) in our SEM model. We chose to eliminate these group of signatures (genes) from the GATA2-related signatures. To provide an unbiased assessment of such analysis, we implemented a two-class bootstrap simulation method "elimination with replacement and elimination without replacement" (Figure 3).

In an elimination without replacement bootstrap analysis, it randomly eliminated the same number of signatures from this originate GATA2-related signatures, then re-calculate the T-score

and re-evaluate the SEM model. In the paper, we suggest a 1,000 round of simulation to provide an empirical distribution for any non-parametric statistics test. On the other hand, in the elimination with replacement bootstrap analysis, after randomly eliminating the same number of signatures from this originate GATA2-related signatures, we replace the same number of "irrelevant" signatures back to the "shrunken" list. Then, we re-calculate the T-score and re-evaluate the SEM model, we also suggest a 1000 round of simulation to provide an empirical distribution for any non-parametric statistics test.

The elimination without replacement simulation was used to test whether a regulator has any impact on our factor (i.e. GATA2) in term of function association; and the elimination with replacement simulation was used to rule out the possibility that the number of downstream signatures of a factor (i.e. GATA2) has any impact on its function. Both empirical distributions serve as the null hypothesis for the statistical testing.

Gene list preparation

The microarray gene expression data was analyzed using The Partek Genomics Suite 7.17 software (Partek Inc., St. Louis, MO). The Robust Multichip Analysis (RMA) algorithm with quantile for normalization and log2 transformation was applied to generate gene expression values of all samples. The one-way analysis of variance (ANOVA) model was used to compare expression profiles from different groups. Differentially expressed genes (DEGs) were identified using the filters of ANOVA unadjusted p value < 0.01 and absolute fold change >1.3.

The published GATA2 occupancy information GEO accession: GSE40659 (Rubel et al. 2016) was first lifted from mm9 to mm10 genome assembly and then annotated by HOMER (Heinz et al. 2010) for the nearby genes. The obtained GATA2 ChIP-seq targets were mapped to the GATA2 signature from microarray data to identify the putative GATA2 direct downstream targets (GATA2 direct signature - Supplemental Table 1). The criteria used to selected GATA2 ChIP-seq targets was GATA2 binding at immediate promoter regions (+/-2kb of TSS).

The main steps to follow the use case example

Step 1. <u>To get the T-score</u>: Users can launch the App and import the 634 genes list (Supplemental Table 1) and HumanArray4Shiny comes with the App. By clicking the green "Go" button, the corresponding T score will then be calculated and can be download (shown in Supplemental Figure 1). We also provided this calculated T-score in Supplemental Table 2.

Step 2. <u>To construct the dataset</u>: Users need to open the _sampleDAT.txt under the "app_installation_dir/dataSEM/", i.e. /Users/li11/myGit/SEMIPs/dataSEM, append the new T - Score column from step 1 and name the header accordingly. We use "GATA2 Direct" in this use case. Please save the new file as "app_installation_dir/dataSEM/sampleDAT.txt".

Step 3. <u>To run the SEM model</u>: Users need to re-launch the app. Under the SEM tab, from the drop-down list select "GATA2 Direct", "PGR_act_FC13_P01", and "SOX17_lev" as show in Supplemental Figure 2. Then the structural equation model will be fitted accordingly. User can

download the 3-node SEM image as well as the model fitting details as shown in Supplemental Figure 2.

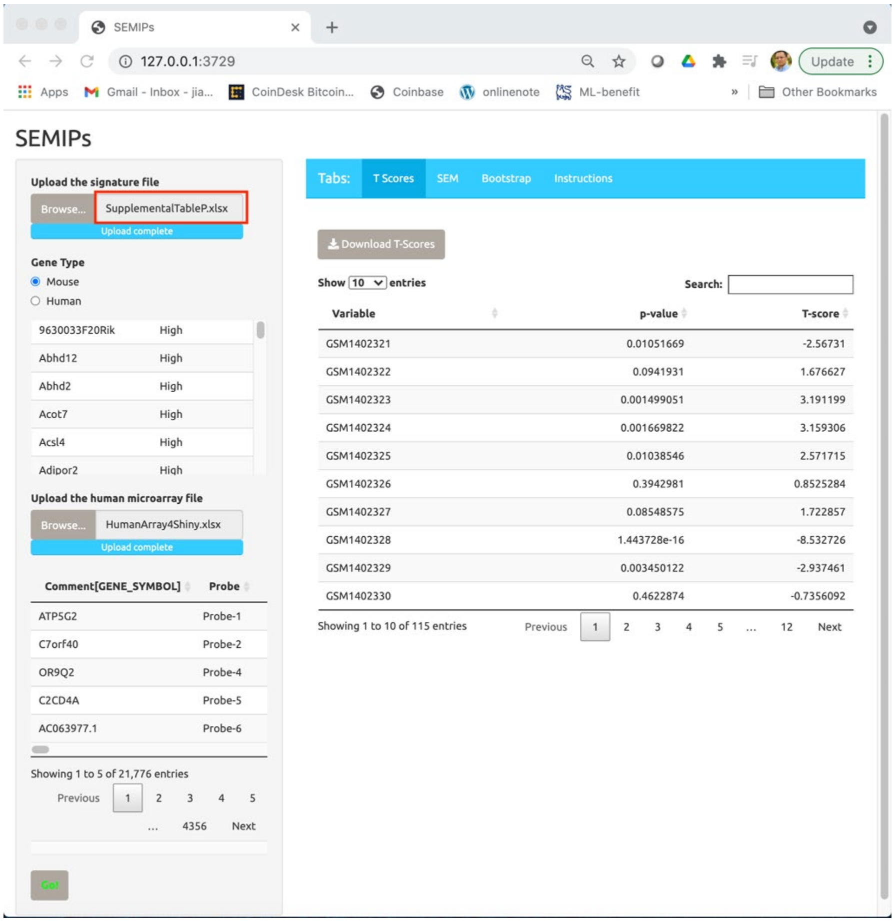

Supplementary Figure 1. An illustration for using the App to calculate T-score for Supplemental Table 1.

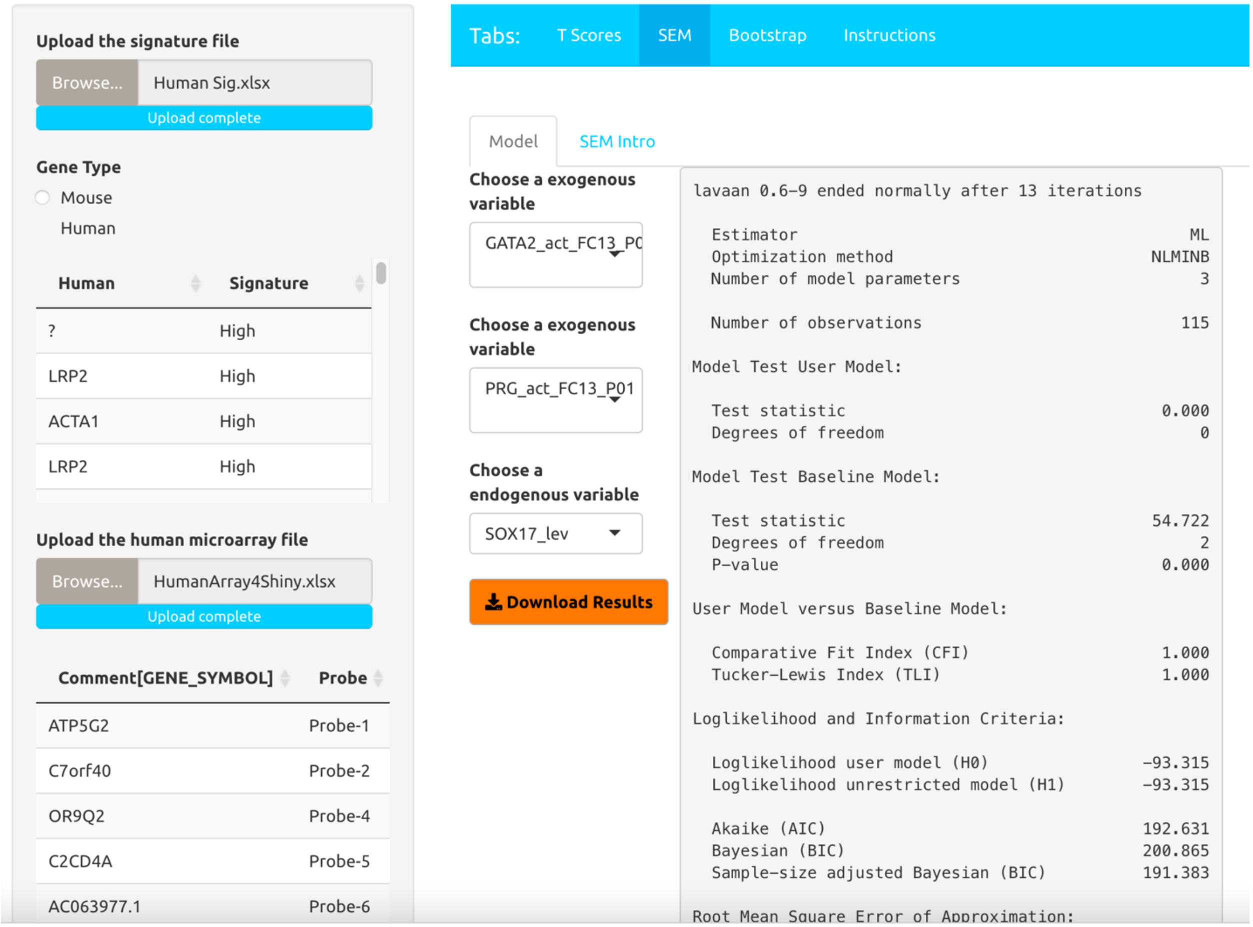

Supplementary Figure 2. An illustration for using the App to fit the structural equation model for Supplemental Table 2 (GATA2 direct gene list). The fitting statistics can be downloaded by clicking the "Download Results" button.

References

Heinz, S., et al. (2010). "Simple combinations of lineage-determining transcription factors prime cis-regulatory elements required for macrophage and B cell identities." Mol Cell **38**(4): 576-589.

Rubel, C. A., et al. (2016). "A Gata2-Dependent Transcription Network Regulates Uterine Progesterone Responsiveness and Endometrial Function." Cell Rep **17**(5): 1414-1425.
